# Bis[μ-2-(2,4-difluoro­phen­yl)-1,3-bis­(1*H*-1,2,4-triazol-1-yl)propan-2-olato]dicopper(II) bis­(perchlorate)

**DOI:** 10.1107/S1600536810008512

**Published:** 2010-03-10

**Authors:** Zhi-Rong Luo, Fei-Long Hu, Yue Zhuang, Xian-Hong Yin, Qiao-Lan Wu

**Affiliations:** a Department of Chemistry, Guangxi University for Nationalities, Nanning 530006, People’s Republic of China

## Abstract

The title complex, [Cu_2_(C_13_H_11_F_2_N_6_O)_2_](ClO_4_)_2_, which was hydro­thermally synthesized, contains a binuclear copper cluster (2 symmetry) with a Cu_2_O_2_ rhombus [Cu—O = 1.927 (2) Å] formed by donation of two O atoms from two chelate rings. The tridentate function of each ligand is completed by two N atoms coordinated to the two Cu^II^ atoms [Cu—N = 1.933 (2) Å]. The separation distance of two Cu^II^ atoms in a cluster is 2.988 (1) Å. The dihedral angle between the six-membered chelate rings is 2.13 (9)°. The perchlorate counter-anion is disordered over two sites in a 0.58 (10):0.42 (10) ratio.

## Related literature

For the use of 1,2,4-triazole and its derivatives in coordination chemistry, see: Haasnoot *et al.* (2000 ▶); Zhao *et al.* (2007 ▶). For 1,2,4-triazole as a bridging ligand, see: Liu *et al.* (2003 ▶); Park *et al.* (2006 ▶); Yi *et al.* (2004 ▶); Garcia *et al.* (2005 ▶).
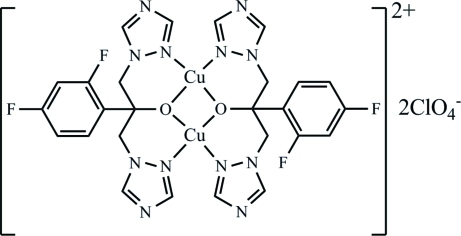


## Experimental

### 

#### Crystal data

[Cu_2_(C_13_H_11_F_2_N_6_O)_2_](ClO_4_)_2_
*M* *_r_* = 936.54
Orthorhombic, 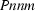

*a* = 15.4464 (18) Å
*b* = 7.9532 (10) Å
*c* = 14.2407 (15) Å
*V* = 1749.4 (4) Å^3^
*Z* = 2
Mo *K*α radiation
μ = 1.46 mm^−1^
*T* = 298 K
0.49 × 0.45 × 0.43 mm

#### Data collection

Bruker SMART 1000 diffractometer
Absorption correction: multi-scan (*SADABS*; Sheldrick, 1996 ▶) *T*_min_ = 0.534, *T*_max_ = 0.572
7867 measured reflections
1618 independent reflections
1169 reflections with *I* > 2σ(*I*)
*R*_int_ = 0.024

#### Refinement

*R*[*F* ^2^ > 2σ(*F* ^2^)] = 0.040
*wR*(*F* ^2^) = 0.129
*S* = 1.09
1618 reflections
162 parameters
H-atom parameters constrained
Δρ_max_ = 0.55 e Å^−3^
Δρ_min_ = −0.48 e Å^−3^

### 

Data collection: *SMART* (Siemens, 1996 ▶); cell refinement: *SAINT* (Siemens, 1996 ▶); data reduction: *SAINT*; program(s) used to solve structure: *SHELXS97* (Sheldrick, 2008 ▶); program(s) used to refine structure: *SHELXL97* (Sheldrick, 2008 ▶); molecular graphics: *SHELXTL* (Sheldrick, 2008 ▶); software used to prepare material for publication: *SHELXTL*.

## Supplementary Material

Crystal structure: contains datablocks I, global. DOI: 10.1107/S1600536810008512/kp2237sup1.cif

Structure factors: contains datablocks I. DOI: 10.1107/S1600536810008512/kp2237Isup2.hkl

Additional supplementary materials:  crystallographic information; 3D view; checkCIF report

## Figures and Tables

**Fig. 1.**
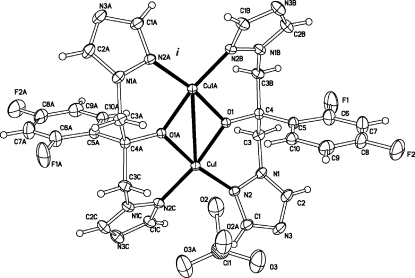
The structure of the title compound and the atom-numbering scheme. The H atoms have been omitted for clarity. [Symmetry code: (i) -x, 1-y, -z.]

**Fig. 2.**
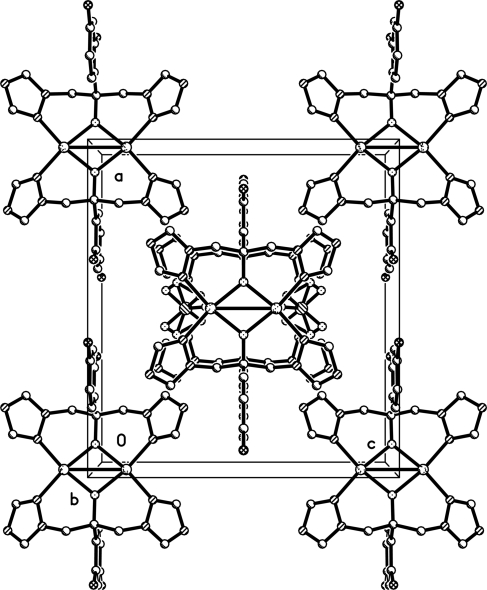
The packing diagram of the title compound.

**Fig. 3.**
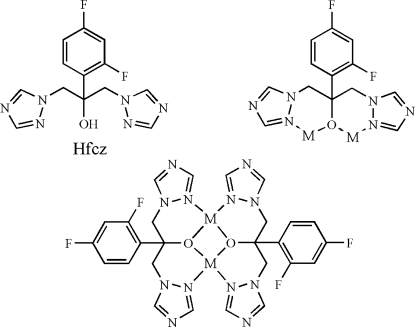
The structure of Hfcz, the distinct binding mode of the ligand and the title compound.

## supplementary crystallographic information

### Comment

1,2,4-triazole and its derivatives, being used in pharmaceuticals and agricultural chemicals, have attracted ever-increasing attention in coordination chemistry (Haasnoot *et al.*, 2000, Zhao *et al.*, 2007). The coordination versatility of 1, 2, 4-triazole as a reliable bridging ligand, i.e. adopting diverse binding fashions upon metal complexation, will be responsible for structural diversity of the resulting coordination frameworks (Liu *et al.*, 2003, Park *et al.*, 2006, Yi *et al.*, 2004, Garcia *et al.*, 2005). Now, we present the synthesis and structure analysis of the title Hflu complex derived from bis-fluconazolato-di-copper diperchlorate. The distinct binding modes of the ligands (Scheme 1) and the coordination preferences of the metal ion are discussed. The title binuclear copper(II) compound, *C*_26_H_22_Cl_2_Cu_2_F_4_N_12_O_10_, reveals a centro-symmetric arrangement. The coordination of Cu1 is achieved by 1, 2, 4-triazole-N and flu-O ligands (Fig. 1); two oxygen atoms of fluconazole molecule form rhombus with two copper atoms with Cu-O1 bond of 1.927 (2) Å, forming a Z-style structure. The perchlorate ion is not coordinated to Cu^II^ atom. It take a part in formation of an ornament of 'palace lantern-style'geometry (Fig. 2). The perchlorate anions adopt two crystallographic orientations in the crystal lattice with occupancy factors of 0.576 and 0.424. The dihedral angle between two 1, 2, 4-triazole groups in the same fluconazole ligand is 68.25 (9) ° and the dihedral angle between two ipso-lateral 1, 2, 4-triazole groups in the same molecular binuclear copper cluster is 68.25 (9) ° (Fig. 1). In order to achieve the optimum coordination, fluconazole molecules were adjusted into epsilon-type arrangement.

### Experimental

A methanolic solution (15 ml) containing the Hflu ligand (0.5 mmol, 0.153 g) was added dropwise to a water solution (10 ml) containing Cu(ClO_4_)_2_ (0.5 mmol, 0.131 g). After stirring for 4 h, the solution was filtered. The filtered solution were evaporated for several days in the air and obtained deep-blue block-shaped crystals that was suitable for single-crystal X-ray diffraction (yield: 65.6% based on the ligand). Anal calc (%). for *C*_26_H_22_Cl_2_Cu_2_F_4_N_12_O_10_: H, 2.37; C, 33.34; N, 17.95. Found: H, 2.45; C,33.41; N, 17.81.

### Refinement

H atoms were positioned geometrically and refined as riding atoms, with C—H = 0.93 Å and *U*_iso_(H) = 1.2*U*_eq_(C) for aromatic H atoms, C—H = 0.97 Å and *U*_iso_(H) = 1.2*U*_eq_(C) for methylene H atoms. The thermalfactors being set 1.5 times of their carrier atoms.

### Crystal data

**Table:**

[Cu_2_(C_13_H_11_F_2_N_6_O)_2_](ClO_4_)_2_	*D*_x_ = 1.778 Mg m^−^^3^
*M**_r_* = 936.54	Mo *K*α radiation, λ = 0.71073 Å
Orthorhombic, *P**n**n**m*	Cell parameters from 3901 reflections
*a* = 15.4464 (18) Å	θ = 2.6–26.9°
*b* = 7.9532 (10) Å	µ = 1.46 mm^−^^1^
*c* = 14.2407 (15) Å	*T* = 298 K
*V* = 1749.4 (4) Å^3^	Block, blue
*Z* = 2	0.49 × 0.45 × 0.43 mm
*F*(000) = 940	

### Data collection

**Table:**

Bruker SMART 1000 diffractometer	1618 independent reflections
Radiation source: fine-focus sealed tube	1169 reflections with *I* > 2σ(*I*)
graphite	*R*_int_ = 0.024
phi and ω scans	θ_max_ = 25.0°, θ_min_ = 2.0°
Absorption correction: multi-scan (*SADABS*; Sheldrick, 1996)	*h* = −16→18
*T*_min_ = 0.534, *T*_max_ = 0.572	*k* = −9→9
7867 measured reflections	*l* = −16→13

### Refinement

**Table:**

Refinement on *F*^2^	Primary atom site location: structure-invariant direct methods
Least-squares matrix: full	Secondary atom site location: difference Fourier map
*R*[*F*^2^ > 2σ(*F*^2^)] = 0.040	Hydrogen site location: inferred from neighbouring sites
*wR*(*F*^2^) = 0.129	H-atom parameters constrained
*S* = 1.09	*w* = 1/[σ^2^(*F*_o_^2^) + (0.0552*P*)^2^ + 3.7317*P*] where *P* = (*F*_o_^2^ + 2*F*_c_^2^)/3
1618 reflections	(Δ/σ)_max_ = 0.001
162 parameters	Δρ_max_ = 0.55 e Å^−^^3^
0 restraints	Δρ_min_ = −0.48 e Å^−^^3^

### Special details

**Table:**

Geometry. All esds (except the esd in the dihedral angle between two l.s. planes) are estimated using the full covariance matrix. The cell esds are taken into account individually in the estimation of esds in distances, angles and torsion angles; correlations between esds in cell parameters are only used when they are defined by crystal symmetry. An approximate (isotropic) treatment of cell esds is used for estimating esds involving l.s. planes.
Refinement. Refinement of F^2^ against ALL reflections. The weighted R-factor wR and goodness of fit S are based on F^2^, conventional R-factors R are based on F, with F set to zero for negative F^2^. The threshold expression of F^2^ > 2sigma(F^2^) is used only for calculating R-factors(gt) etc. and is not relevant to the choice of reflections for refinement. R-factors based on F^2^ are statistically about twice as large as those based on F, and R- factors based on ALL data will be even larger.

### Fractional atomic coordinates and isotropic or equivalent isotropic displacement parameters (Å^2^)

**Table:**

	*x*	*y*	*z*	*U*_iso_*/*U*_eq_	Occ. (<1)
Cu1	0.0000	0.5000	0.10491 (4)	0.0387 (3)	
Cl1	0.0000	1.0000	0.1949 (2)	0.0786 (7)	
F1	0.3498 (3)	0.3943 (7)	0.0000	0.112 (2)	
F2	0.3994 (4)	0.9719 (7)	0.0000	0.110 (2)	
N1	0.1704 (2)	0.4153 (4)	0.1725 (2)	0.0412 (8)	
N2	0.0936 (2)	0.4883 (4)	0.1947 (2)	0.0394 (8)	
N3	0.1944 (3)	0.5620 (5)	0.2995 (3)	0.0595 (11)	
O1	0.0783 (2)	0.4823 (5)	0.0000	0.0339 (8)	
O2	0.009 (3)	0.8525 (18)	0.126 (2)	0.097 (7)	0.58 (10)
O3	0.0787 (17)	1.020 (6)	0.232 (3)	0.110 (11)	0.58 (10)
O2'	0.047 (4)	0.884 (6)	0.151 (3)	0.106 (13)	0.42 (10)
O3'	0.055 (3)	1.093 (8)	0.261 (3)	0.109 (11)	0.42 (10)
C1	0.1119 (3)	0.5755 (6)	0.2716 (3)	0.0503 (11)	
H1	0.0711	0.6402	0.3033	0.060*	
C2	0.2285 (3)	0.4627 (6)	0.2355 (3)	0.0546 (12)	
H2	0.2863	0.4298	0.2346	0.066*	
C3	0.1787 (3)	0.3154 (5)	0.0873 (3)	0.0433 (10)	
H3A	0.1359	0.2260	0.0878	0.052*	
H3B	0.2357	0.2641	0.0853	0.052*	
C4	0.1658 (3)	0.4248 (7)	0.0000	0.0352 (12)	
C5	0.2277 (4)	0.5736 (7)	0.0000	0.0395 (13)	
C6	0.3173 (4)	0.5544 (9)	0.0000	0.0640 (19)	
C7	0.3745 (5)	0.6848 (12)	0.0000	0.079 (2)	
H7	0.4339	0.6656	0.0000	0.095*	
C8	0.3431 (5)	0.8399 (11)	0.0000	0.069 (2)	
C9	0.2571 (5)	0.8714 (9)	0.0000	0.0640 (19)	
H9	0.2369	0.9815	0.0000	0.077*	
C10	0.1993 (4)	0.7384 (7)	0.0000	0.0448 (14)	
H10	0.1402	0.7604	0.0000	0.054*	

### Atomic displacement parameters (Å^2^)

**Table:**

	*U*^11^	*U*^22^	*U*^33^	*U*^12^	*U*^13^	*U*^23^
Cu1	0.0307 (4)	0.0620 (5)	0.0232 (3)	−0.0056 (3)	0.000	0.000
Cl1	0.0398 (9)	0.0638 (12)	0.132 (2)	0.0021 (9)	0.000	0.000
F1	0.049 (3)	0.078 (3)	0.208 (7)	0.022 (2)	0.000	0.000
F2	0.115 (4)	0.105 (4)	0.111 (4)	−0.075 (3)	0.000	0.000
N1	0.0453 (19)	0.0394 (18)	0.0390 (18)	−0.0007 (16)	−0.0119 (15)	0.0017 (16)
N2	0.0415 (18)	0.049 (2)	0.0276 (16)	−0.0035 (16)	−0.0047 (14)	0.0001 (15)
N3	0.061 (2)	0.064 (3)	0.054 (2)	−0.004 (2)	−0.022 (2)	−0.012 (2)
O1	0.0277 (18)	0.047 (2)	0.0268 (18)	0.0006 (16)	0.000	0.000
O2	0.073 (13)	0.066 (6)	0.153 (12)	−0.002 (6)	0.008 (10)	−0.014 (6)
O3	0.068 (8)	0.101 (17)	0.162 (17)	0.005 (9)	−0.027 (9)	−0.003 (15)
O2'	0.08 (2)	0.087 (15)	0.152 (17)	0.024 (16)	0.009 (16)	−0.013 (13)
O3'	0.078 (14)	0.10 (2)	0.151 (15)	0.002 (14)	−0.022 (11)	−0.018 (13)
C1	0.054 (3)	0.056 (3)	0.041 (2)	0.000 (2)	−0.010 (2)	−0.006 (2)
C2	0.049 (3)	0.059 (3)	0.056 (3)	0.002 (2)	−0.021 (2)	−0.004 (2)
C3	0.051 (2)	0.036 (2)	0.042 (2)	0.0042 (19)	−0.007 (2)	0.0003 (18)
C4	0.031 (3)	0.033 (3)	0.041 (3)	0.000 (2)	0.000	0.000
C5	0.035 (3)	0.039 (3)	0.045 (3)	0.001 (3)	0.000	0.000
C6	0.047 (4)	0.059 (4)	0.086 (5)	0.000 (3)	0.000	0.000
C7	0.047 (4)	0.089 (7)	0.101 (7)	−0.015 (4)	0.000	0.000
C8	0.068 (5)	0.067 (5)	0.071 (5)	−0.036 (4)	0.000	0.000
C9	0.080 (5)	0.045 (4)	0.067 (5)	−0.010 (4)	0.000	0.000
C10	0.048 (3)	0.042 (3)	0.045 (3)	−0.002 (3)	0.000	0.000

### Geometric parameters (Å, °)

**Table:**

Cu1—O1^i^	1.927 (2)	N3—C1	1.340 (6)
Cu1—O1	1.927 (2)	O1—C4	1.427 (6)
Cu1—N2	1.933 (3)	O1—Cu1^i^	1.927 (2)
Cu1—N2^ii^	1.933 (3)	C1—H1	0.9300
Cu1—Cu1^i^	2.9880 (13)	C2—H2	0.9300
Cl1—O2'	1.33 (2)	C3—C4	1.531 (5)
Cl1—O2'^iii^	1.33 (2)	C3—H3A	0.9700
Cl1—O3^iii^	1.336 (12)	C3—H3B	0.9700
Cl1—O3	1.336 (12)	C4—C5	1.522 (8)
Cl1—O3'	1.47 (3)	C4—C3^iv^	1.531 (5)
Cl1—O3'^iii^	1.47 (3)	C5—C10	1.382 (8)
Cl1—O2	1.53 (2)	C5—C6	1.392 (9)
Cl1—O2^iii^	1.53 (2)	C6—C7	1.364 (10)
F1—C6	1.368 (9)	C7—C8	1.326 (12)
F2—C8	1.364 (8)	C7—H7	0.9300
N1—C2	1.324 (5)	C8—C9	1.352 (11)
N1—N2	1.358 (5)	C9—C10	1.383 (9)
N1—C3	1.456 (5)	C9—H9	0.9300
N2—C1	1.326 (5)	C10—H10	0.9300
N3—C2	1.316 (6)		
			
O1^i^—Cu1—O1	78.33 (16)	C1—N2—Cu1	132.8 (3)
O1^i^—Cu1—N2	170.37 (12)	N1—N2—Cu1	121.3 (2)
O1—Cu1—N2	92.30 (13)	C2—N3—C1	102.9 (4)
O1^i^—Cu1—N2^ii^	92.30 (13)	C4—O1—Cu1^i^	128.15 (10)
O1—Cu1—N2^ii^	170.37 (12)	C4—O1—Cu1	128.15 (10)
N2—Cu1—N2^ii^	97.2 (2)	Cu1^i^—O1—Cu1	101.67 (16)
O1^i^—Cu1—Cu1^i^	39.16 (8)	N2—C1—N3	113.9 (4)
O1—Cu1—Cu1^i^	39.16 (8)	N2—C1—H1	123.0
N2—Cu1—Cu1^i^	131.42 (10)	N3—C1—H1	123.0
N2^ii^—Cu1—Cu1^i^	131.42 (10)	N3—C2—N1	111.7 (4)
O2'—Cl1—O2'^iii^	124 (3)	N3—C2—H2	124.2
O2'—Cl1—O3^iii^	127 (2)	N1—C2—H2	124.2
O2'^iii^—Cl1—O3^iii^	76.9 (12)	N1—C3—C4	110.8 (3)
O2'—Cl1—O3	76.9 (12)	N1—C3—H3A	109.5
O2'^iii^—Cl1—O3	127 (2)	C4—C3—H3A	109.5
O3^iii^—Cl1—O3	133 (4)	N1—C3—H3B	109.5
O2'—Cl1—O3'	110.0 (11)	C4—C3—H3B	109.5
O2'^iii^—Cl1—O3'	105.2 (13)	H3A—C3—H3B	108.1
O3^iii^—Cl1—O3'	109 (3)	O1—C4—C5	110.2 (4)
O3—Cl1—O3'	33.1 (6)	O1—C4—C3^iv^	107.8 (3)
O2'—Cl1—O3'^iii^	105.2 (13)	C5—C4—C3^iv^	111.1 (3)
O2'^iii^—Cl1—O3'^iii^	110.0 (11)	O1—C4—C3	107.8 (3)
O3^iii^—Cl1—O3'^iii^	33.1 (6)	C5—C4—C3	111.1 (3)
O3—Cl1—O3'^iii^	109 (3)	C3^iv^—C4—C3	108.6 (4)
O3'—Cl1—O3'^iii^	100 (2)	C10—C5—C6	114.8 (6)
O2'—Cl1—O2	28 (2)	C10—C5—C4	122.6 (5)
O2'^iii^—Cl1—O2	106.2 (16)	C6—C5—C4	122.6 (6)
O3^iii^—Cl1—O2	104.1 (11)	C7—C6—F1	118.0 (7)
O3—Cl1—O2	105.3 (11)	C7—C6—C5	124.1 (7)
O3'—Cl1—O2	138.4 (14)	F1—C6—C5	117.8 (6)
O3'^iii^—Cl1—O2	94.2 (13)	C8—C7—C6	118.0 (7)
O2'—Cl1—O2^iii^	106.2 (16)	C8—C7—H7	121.0
O2'^iii^—Cl1—O2^iii^	28 (2)	C6—C7—H7	121.0
O3^iii^—Cl1—O2^iii^	105.3 (11)	C7—C8—C9	122.2 (7)
O3—Cl1—O2^iii^	104.1 (11)	C7—C8—F2	118.8 (8)
O3'—Cl1—O2^iii^	94.2 (13)	C9—C8—F2	119.0 (8)
O3'^iii^—Cl1—O2^iii^	138.4 (14)	C8—C9—C10	119.5 (7)
O2—Cl1—O2^iii^	101 (2)	C8—C9—H9	120.2
C2—N1—N2	108.2 (3)	C10—C9—H9	120.2
C2—N1—C3	131.3 (4)	C5—C10—C9	121.4 (6)
N2—N1—C3	120.3 (3)	C5—C10—H10	119.3
C1—N2—N1	103.3 (3)	C9—C10—H10	119.3
			
C2—N1—N2—C1	−0.3 (4)	Cu1—O1—C4—C5	−99.6 (3)
C3—N1—N2—C1	−176.2 (4)	Cu1^i^—O1—C4—C3^iv^	−21.8 (5)
C2—N1—N2—Cu1	163.9 (3)	Cu1—O1—C4—C3^iv^	138.9 (3)
C3—N1—N2—Cu1	−12.0 (5)	Cu1^i^—O1—C4—C3	−138.9 (3)
O1^i^—Cu1—N2—C1	121.8 (9)	Cu1—O1—C4—C3	21.8 (5)
O1—Cu1—N2—C1	135.0 (4)	N1—C3—C4—O1	−65.7 (4)
N2^ii^—Cu1—N2—C1	−46.9 (4)	N1—C3—C4—C5	55.2 (5)
Cu1^i^—Cu1—N2—C1	133.1 (4)	N1—C3—C4—C3^iv^	177.8 (2)
O1^i^—Cu1—N2—N1	−37.0 (10)	O1—C4—C5—C10	0.0
O1—Cu1—N2—N1	−23.8 (3)	C3^iv^—C4—C5—C10	119.5 (3)
N2^ii^—Cu1—N2—N1	154.3 (3)	C3—C4—C5—C10	−119.5 (3)
Cu1^i^—Cu1—N2—N1	−25.7 (3)	O1—C4—C5—C6	180.0
O1^i^—Cu1—O1—C4	−164.6 (5)	C3^iv^—C4—C5—C6	−60.5 (3)
N2—Cu1—O1—C4	17.6 (4)	C3—C4—C5—C6	60.5 (3)
N2^ii^—Cu1—O1—C4	−151.2 (8)	C10—C5—C6—C7	0.0
Cu1^i^—Cu1—O1—C4	−164.6 (5)	C4—C5—C6—C7	180.0
O1^i^—Cu1—O1—Cu1^i^	0.0	C10—C5—C6—F1	180.0
N2—Cu1—O1—Cu1^i^	−177.77 (17)	C4—C5—C6—F1	0.0
N2^ii^—Cu1—O1—Cu1^i^	13.5 (10)	F1—C6—C7—C8	180.0
N1—N2—C1—N3	−0.3 (5)	C5—C6—C7—C8	0.0
Cu1—N2—C1—N3	−161.8 (3)	C6—C7—C8—C9	0.0
C2—N3—C1—N2	0.8 (6)	C6—C7—C8—F2	180.0
C1—N3—C2—N1	−0.9 (6)	C7—C8—C9—C10	0.0
N2—N1—C2—N3	0.8 (5)	F2—C8—C9—C10	180.0
C3—N1—C2—N3	176.1 (4)	C6—C5—C10—C9	0.0
C2—N1—C3—C4	−110.2 (5)	C4—C5—C10—C9	180.0
N2—N1—C3—C4	64.5 (5)	C8—C9—C10—C5	0.0
Cu1^i^—O1—C4—C5	99.6 (3)		

Symmetry codes: (i) −*x*, −*y*+1, −*z*; (ii) −*x*, −*y*+1, *z*; (iii) −*x*, −*y*+2, *z*; (iv) *x*, *y*, −*z*.
